# A Novel Prognostic Nomogram and Risk Classification System for Predicting Cancer-Specific Survival of Postoperative Fibrosarcoma Patients: A Large Cohort Retrospective Study

**DOI:** 10.1155/2022/7831001

**Published:** 2022-08-27

**Authors:** Chao Huang, Zhangheng Huang, Zongke Zhou

**Affiliations:** Department of Orthopedics, West China Hospital of Sichuan University, No. 37 Guoxue Alley, Wuhou District, Chengdu 610041, Sichuan, China

## Abstract

**Background:**

Fibrosarcoma (FS) is a typically invasive sarcoma formed by fibroblasts and collagen fibers. Currently, the standard treatment for FS is the surgical resection, but the high recurrence rate and poor prognosis limit the benefits of postoperative patients. Exploring what factors affect the benefit of postoperative patients is significant for guiding the implementation of surgical resection. Therefore, this study aims to construct a novel nomogram to predict the cancer-specific survival (CSS) of postoperative fibrosarcoma (POFS) patients.

**Methods:**

The included patients were randomly assigned to the training and validation sets at a ratio of 7 : 3. CSS was indexed as the research endpoint. Firstly, univariate and multivariate Cox regression analyses were used on the training set to determine independent prognostic predictors and build a nomogram for predicting the 1-, 3-, and 5-year CSS of POFS patients. Secondly, the nomogram's discriminative power and prediction accuracy were evaluated by receiver operating characteristic (ROC) and the calibration curve, and a risk classification system for POFS patients was constructed. Finally, the nomogram's clinical utility was evaluated using decision curve analysis (DCA).

**Results:**

Our study included 346 POFS patients, divided into the training (244) and validation sets (102). Multivariate Cox regression analysis demonstrated that tumor size, SEER stage, and tumor grade were independent prognostic predictors of CSS for POFS patients. They were used to create a nomogram. In the training and validation sets, the ROC curve showed that the 1-, 3-, and 5-year area under the curve (AUC) were higher than 0.700, indicating that the nomogram had good reliability and accuracy. DCA also showed that the nomogram has high application value in clinical practice.

**Conclusion:**

The larger tumor size, higher tumor grade, and distant metastasis were independently related to the poor prognosis of POFS patients. The nomogram constructed based on the above variables could accurately predict the 1-, 3-, and 5-year CSS of POFS patients. So, the nomogram and risk classification system we built might help make accurate judgments in clinical practice, optimize patient treatment decisions, maximize postoperative benefits, and ultimately improve the prognosis of POFS patients.

## 1. Introduction

Fibrosarcoma (FS) is a malignant mesenchymal tumor, composed of fibroblasts with variable collagen production [1]. All body parts containing fibrous tissue may be the birthplace of FS, but it is more common in head, neck, trunk, and limbs, accounting for approximately 5% of all soft tissue sarcomas [2]. FS could be divided into adult FS and infantile FS according to age. Infantile FS usually occurs in children under 5 years old. Congenital infantile FS is rare, accounting for less than 1% of all childhood cancers. Infantile FS was defined as a moderately malignant and rarely metastatic tumor by the World Health Organization (WHO), while adult FS was classified as a highly malignant tumor [3–5]. Adult FS accounts for approximately 3.6% of all adult sarcomas and mainly occurs in people between 25 and 79 years old, and the incidence of men and women is roughly the same [5, 6]. Other more common subtypes of FS include dermatofibrosarcoma protuberans (DFSP). DFSP mainly occurs in middle-aged men. Generally, DFSP's growth rate is relatively slow, with a low metastasis rate (<5%), but the local recurrence rate is high (20–50%), especially when the resection is not enough [7, 8].

Although FS is mainly treated by surgical resection, it was reported that 10–20% of patients whose tumors were fully resected will experience recurrence within 5 years, and the prognosis of those patients was worse [9]. Thus, conducting a separate analysis to find the most relevant prognostic factors related to the survival rate of POFS patients and carry out individualized management for postoperative patients to improve the effectiveness of the surgical treatment is necessary. Compared with overall survival (OS), cancer-specific survival (CSS) can provide a closer relationship with tumor-mediated patient prognosis and provide more precise guidance for treating those patients. However, as far as we know, no research has focused on the development of predictive models for CSS in patients with POFS. Therefore, our study aims to find the prognostic predictors related to CSS of POFS patients by analyzing relevant data from the surveillance, epidemiology, and end results (SEER) database and to develop a new nomogram model and a risk classification system to predict the 1-, 3-, and 5-year CSS of POFS patients.

## 2. Methods

### 2.1. Database

Sponsored by the National Cancer Institute, the SEER data set (https://seer.cancer.gov/seerstat/) is a cancer database based on the US population, providing systematic evidence support and valuable direct information for clinicians' practice and medical research. It collected data from 18 registries, nearly 30% of the US population [10]. SEER Stat 8.3.9.2 was used to identify the data of all POFS patients from 1975 to 2016 in SEER database [Incidence-SEER 18 Regs Custom Data (with additional treatment fields), Nov 2018 Sub (1975–2016 varying)]. We obtained access to the SEER database after obtaining permission to access research data files with the reference number 16336-Nov2020. Since the acquired data has no specific personal information disclosed, it does not require the ethics committee's approval and the patient's informed consent. This study was conducted and reported in line with STROCSS 2019 criteria [11].

### 2.2. Patient Selection

The inclusion criteria of our study were as follows: (i) FS is the patient's ICD-O-3 histological type; (ii) complete follow-up data; (iii) primary tumor; (iv) surgery performed, while the exclusion criteria were as follows: (i) it is not the primary tumor; (ii) the information about age, sex, race, marriage, tumor stage, tumor grade, tumor size, surgery, radiotherapy, and chemotherapy was unknown; (iii) survival time is less than one month. Finally, 346 POFS patients were found to be suitable for inclusion in our study.

All included patients were randomly divided into a training set (70%) and a validation set (30%) according to a ratio of 7 : 3. We used the training set to determine independent prognostic predictors and establish the prognostic nomogram for postoperative patients, and the validation set was used to verify the nomogram.

### 2.3. Variable Definitions

Variables included in our study were POFS patients' demographic characteristics (age, race, sex, and marital status), disease characteristics (tumor size, tumor grade, and SEER histological stage), and information on treatment (radiotherapy and chemotherapy). The X-tile software (version 3.6.1) determined the best cut-off values for age and tumor size, and the results showed that the best cut-off values for age were 43 and 71 years, and those for tumor size were 6.4 and 11 cm, respectively (Supplementary File 1) [12]. Sex was divided into male and female, and the race was divided into white, black, and others. Marital status was divided into married and unmarried. Radiotherapy and chemotherapy were divided into yes and no. Tumor grades were divided into grades I, II, III, and IV, and SEER histological stages were classified as local, regional, and distant. Our study's primary endpoint was CSS, which was defined as the time interval between the day of diagnosis and death caused by this tumor alone.

### 2.4. Statistical Analysis

SPSS (version 22.0) and R software (version 4.0.3) was used to do statistical analyses in this study, and a *p*-value of <0.05 was considered statistically significant. First, values were assigned to CSS-related variables (Supplementary File 2). The Kaplan-Meier method was generated to show the statistical difference between the included variables, and univariate Cox regression analysis was performed. Then, the variables with *p*-value <0.05 obtained in the univariate Cox regression analysis were selected and included in the multivariate Cox regression analysis to determine the independent prognostic predictors of CSS for the POFS patients. A nomogram for predicting 1-, 3-, and 5-year CSS was established based on these independent prognostic predictors, and the corresponding point assignments for independent prognostic predictors were also obtained (Supplementary File 3). After that, 1-, 3-, and 5-year calibration curves were established to exhibit the nomogram's correction ability, and a decision curve analysis (DCA) was performed to demonstrate the nomogram's clinical benefits. Meanwhile, receiver operating characteristic (ROC) curves for 1-, 3-, and 5- years CSS were established, and the corresponding area under the curve (AUC) value was used to evaluate the nomogram's discriminative ability. In addition, points assigned to independent prognostic predictors were used to calculate the patients' total points, and the best cut-off value for the total points was obtained using the X-tile software. Then, the enrolled patients were divided into low-, middle-, and high-risk subgroups to create a risk classification system, stratifying the death risk of all POFS patients. Finally, the difference in CSS between the three subgroups was obtained using the Kaplan-Meier method.

## 3. Results

### 3.1. Baseline Characteristics

346 patients with POFS were enrolled in this study and randomly divided into a training set (244, 70%) and a validation set (102, 30%), of which 172 (49.7%) were aged 43–71 years; sex and marital status disparity were not apparent; the majority of patients were white (237, 68.5%); the diameter of the tumor was within 6.4 cm in 62.42% of all patients; besides, patients with low-grade (I-II) tumor accounted for 67.63% of the study population and 77.74% of them were diagnosed as localized metastasis; in addition to surgical treatment for those POFS patients, 11.56% of patients underwent chemotherapy, and 33.24% received radiotherapy (Table 1).

### 3.2. Identification of Prognostic Predictors for CSS

**Figure 1:**
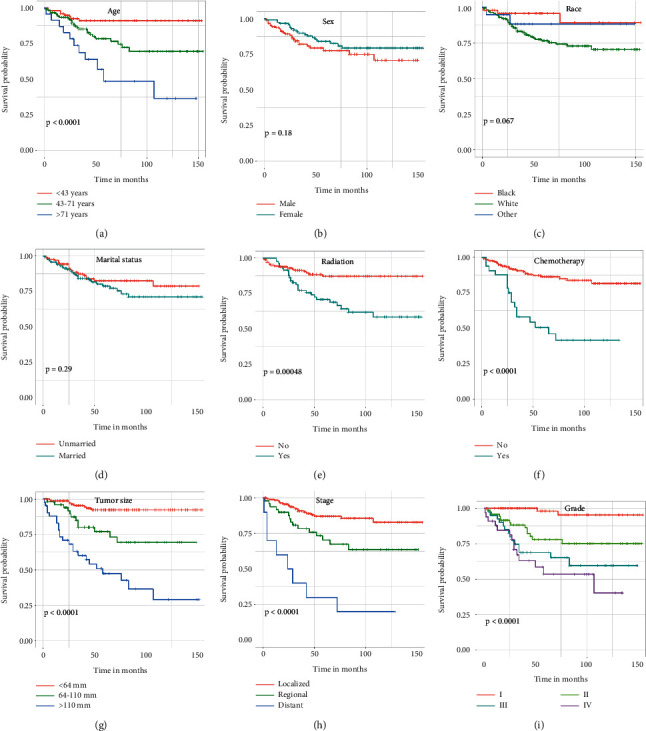
Kaplan–Meier curves of variables were performed for cancer-specific. Survival (CSS) in postoperative fibrosarcoma (POFS) patients. (a) Age, (b) sex, (c) race, (d) marital status, (e) radiation, (f) chemotherapy, (g) tumor size, (h) tumor stage, and (i) tumor grade.

Univariate and multivariate Cox regression analyses were performed to explore independent prognostic predictors of CSS for POFS patients. Age, sex, race, radiotherapy, chemotherapy, tumor size, SEER stage, tumor grade, and marital status were included in univariate Cox regression analysis and the Kaplan-Meier method was performed for CSS in POFS patients (Figure 1). The results of univariate Cox regression analysis revealed that age, race, radiotherapy, chemotherapy, tumor size, tumor grade, and tumor stage were identified as CSS-related variables (*p* < 0.05), while sex and marital status had no significant difference (*p* > 0.05) (Table 2). Then, the multivariate Cox regression analysis was performed to eliminate confounding effects between the above CSS-related variables (*p* < 0.05), and the result showed that tumor size, tumor grade, and SEER stage were identified as independent prognostic predictors of CSS for POFS patients (Table 2). POFS patients with larger tumor size, higher tumor grade, and distant metastasis would be associated with poor CSS.

### 3.3. Establishment and Verification of the Prognostic Nomogram for the CSS

**Figure 2:**
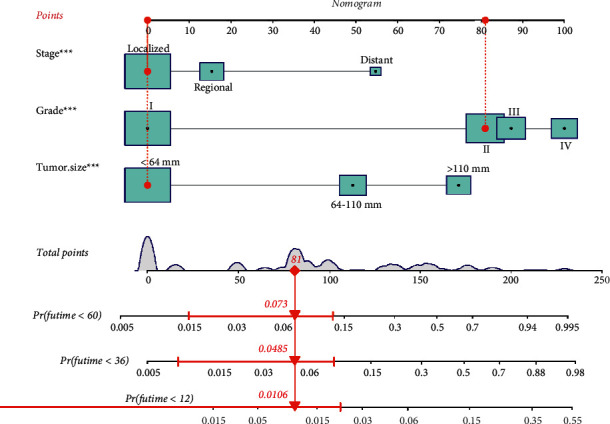
The nomogram model predicts the 1-, 3-, and 5-year CSS of POFS patients.

**Figure 3:**
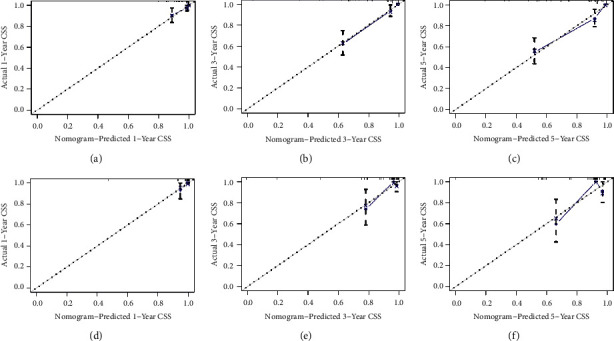
The training and the validation sets' calibration curves in our study. The nomogram's calibration curves for the 1-, 3-, and 5-year CSS prediction of POFS patients in the training set (a–c) and the validation set (d–f).

**Figure 4:**
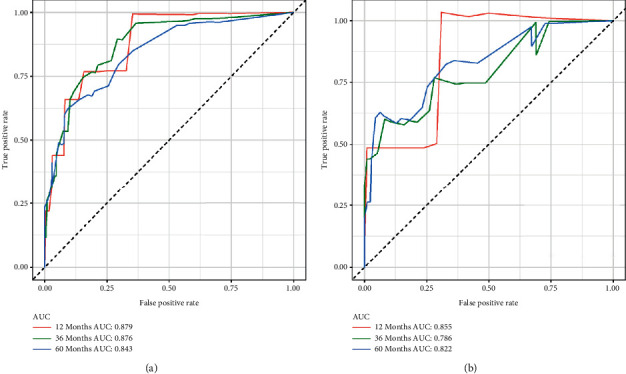
1-, 3-, and 5-year receiver operating characteristic (ROC) curves in the training (a) and validation (b) sets of POFS patients.

To predict the CSS of POFS patients, we developed a nomogram based on all the above independent CSS-related predictors from multivariate Cox regression analysis (Figure 2). The nomogram also endowed each independent prognostic predictor with a point. Adding these points could predict the 1-, 3-, and 5-year CSS of POFS patients. As shown in Figure 2, smaller tumor size, lower tumor grade, and localized metastasis are protective predictors for POFS patients. The poor prognosis of POFS patients included larger tumor size, higher tumor grade, and distant metastasis. The excellent agreement between the predicted results and the actual survival rate of POFS patients was reflected by the 1-, 3-, and 5-year calibration curves (Figure 3). The AUCs for CSS at 1-, 3-, and 5-year in the ROC curve of the training set were 0.879, 0.876, and 0.843, and those in validation set were 0.855, 0.786, and 0.822, respectively (Figure 4), indicating that the nomogram had good reliability and accuracy.

**Figure 5:**
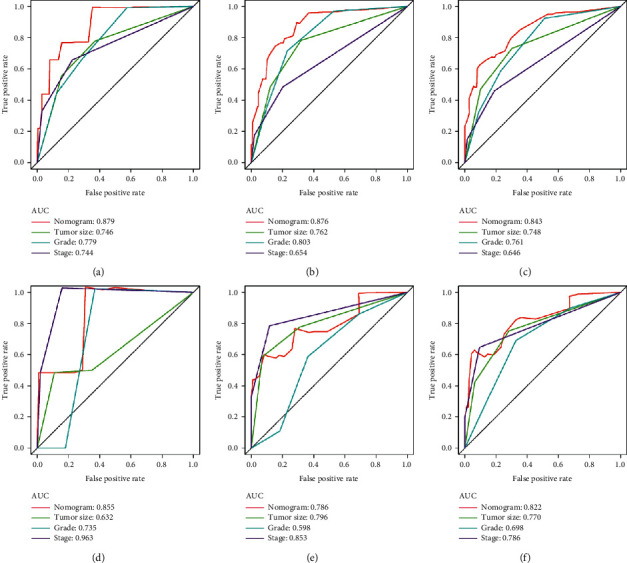
Comparison of the prediction accuracy between the nomogram model and independent predictors in our study. The nomogram and all independent predictors' ROC curves at 1- (a), 3- (b), and 5-year (c) in the training set and at 1- (d), 3- (e), and 5-year (f) in the validation set.

**Figure 6:**
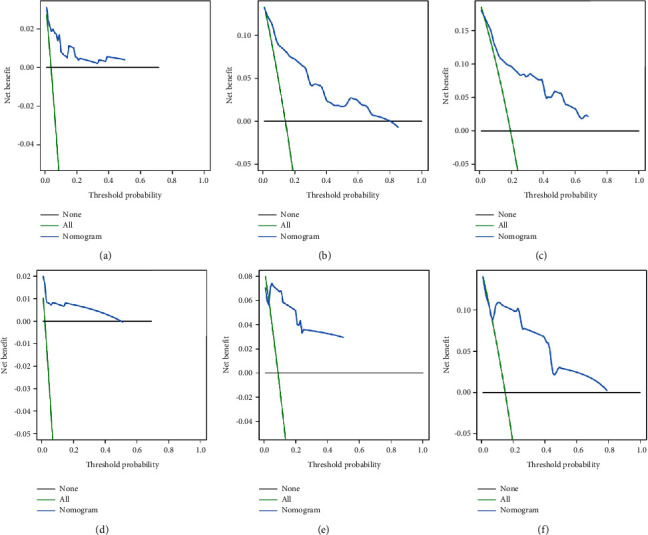
The training and the validation sets' decision curve analysis (DCA) in our study. DCA of the nomogram for predicting the 1- (a), 3- (b), and 5- year (c) CSS in the training set and the 1- (d), 3- (e), and 5- year (f) CSS in the validation set of POFS patients.

Meanwhile, we also compared the prediction accuracy of a single independent prognostic predictor with the nomogram (Figure 5). As shown in Figure 5, the AUCs of each independent prognostic predictor of CSS in training set at 1-, 3-, and 5-year calibration curves were lower than the nomogram, indicating that the nomogram had better prediction accuracy for CSS, while, in the validation set, nomogram's AUCs in the 1- and 3-yearROC curves were not as large as those of some individual independent predictors, which might be related to the small number of postoperative death patients in the 1- and 3-yearROC curves in the validation set (2/6, 33.3% and 8/36, 25%, respectively), but as the follow-up time increased, the AUC of the nomogram gradually increased and showed better prediction accuracy, as shown in Figure 5(f). Besides, the DCA showed that the nomogram had a high clinical application value and could be used as an effective auxiliary tool in clinical practice to maximize the benefit of postoperative patients (Figure 6).

### 3.4. Risk Classification System

**Figure 7:**
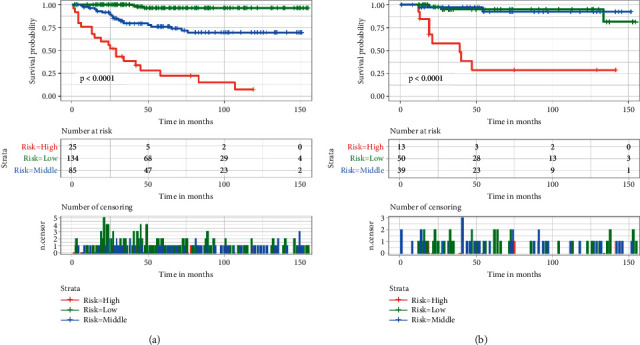
The training and the validation sets' Kaplan–Meier survival analysis in our study. Patients with lower risk scores showed a better prognosis than patients with high-risk scores in the training set (a) and the validation set (b) for the CSS of POFS patients in our cohort retrospective study.

We constructed a disease risk classification system for POFS patients based on the tumor size, tumor grade, and SEER historical stage to further verify the nomogram from different dimensions. And we calculated the patient's total point based on the assignment of the nomogram for the included independent predictors. The best cut-off values for the total point were 87 and 156 using the X-tile software (Supplementary File 1), and the patients were further divided into three different death risk classification subgroups based on the total point: low- (<87), middle- (87–156), and high- (>156) risk subgroups, and a Kaplan-Meier curve was drawn (Figure 7). As shown in Figure 7, whether it is a training set or a validation set, risk classification system can efficiently divide POFS patients into three subgroups with significant differences (*p* < 0.05), indicating that the nomogram has a significant predictive value in the prognosis of the subgroups POFS patients.

## 4. Discussion

FS is defined as fibroblast/myofibroblastic sarcoma and a rare high-grade malignant tumor derived from mesenchymal cells according to the WHO classification of soft tissue sarcoma [9]. The overall 5-year survival rate of FS is about 40–60%, and the recurrence rate is between 12 and 79% [13, 14]. Numerous studies have shown that the unfavorable prognostic factors of FS included the following aspects: (i) large tumor size (>5 cm in diameter); (ii) high histological grade; (iii) deep tumor; (iv) a decrease of collagen fibers; (v) massive mitotic phase (>20/10 high power field); and (vi) massive tissue necrosis (>50%) [6, 9, 13]. It was not until the last two years that articles about the prediction of the survival rate of FS were reported. In 2020, Xiang et al. established a nomogram based on 663 patients with FS through the SEER database, which showed that surgery, sex, tumor size, SEER stage, and pathological grade were the independent prognostic predictors of OS and CSS of FS patients [15]. Subsequently, Yang et al. constructed a novel nomogram based on 357 elderly FS patients, which showed that those patients' OS was related to age, tumor grade, surgery, chemotherapy, and SEER stage [16]. Surgical resection is essential for the treatment of FS. However, recurrence would occur in patients with fully resected tumors, causing poor progress. Thus, exploring prognostic factors that affect the survival benefits of surgical patients is necessary and will help evaluate and determine whether the patients need surgery and ensure the maximum benefits of surgery to FS patients.

Nomogram is a model for multi-index joint diagnosis or prediction of disease incidence or progression and is widely used in tumor diseases. The advantage of the nomogram is to simplify the complex statistical prediction model involving a large number of variables into a single short numerical estimation model to predict the probability of an event [17]. Each independent risk factor included in the model is assigned a value to evaluate the impact of the factor on the occurrence of the event. Therefore, when predicting the survival rate of cancer patients, nomograms can help clinicians optimize treatment plans for specific individual variables. Meanwhile, compared with the traditional American Joint Committee on Cancer (AJCC) tumor-node-metastasis (TNM) staging system, the nomogram has better performance and has the unique advantage of being a tailor-made survival prediction model.

Our study selected 346 POFS patients from the SEER database, and tumor size, SEER stage, and tumor grade were identified as independent prognostic predictors for CSS of POFS patients according to the multivariate Cox regression analysis. Therefore, we established a nomogram model for predicting the 1-, 3-, and 5-year CSS of POFS patients. Both the training and the validation sets showed that the nomogram had reasonable discrimination. There is no significant deviation between the actual survival rate and the predicted survival rate. The nomogram can be used as a practical clinical prediction tool and applied in the clinic. Besides, the constructed risk classification system can effectively divide the patients in the training and validation sets into low-, middle-, and high-risk subgroups with significant differences (*p* < 0.05), which can achieve better patient risk differentiation and effective intervention treatment. Currently, the specific etiology of FS has not yet been definitively concluded, and FS is mainly treated by surgical resection [7]. The principle of surgical resection is radical resection or barrier resection, with negative margins in all directions. The surgical procedure varies according to the location, size, and degree of the tumor malignancy. If the tumor was located in the extremities, some bone might need to be removed and replaced with a prosthesis or bone graft. When the tumor involves the nerves and blood vessels of the limb, the limb must be amputated. However, surgical treatment has major drawbacks for the removal of giant tumors. When the surgical margin is not complete, the residual lesions will lead to local tumor recurrence, further leading to tumor metastasis and a poor prognosis for patients after FS. In a single-center study carried out by Bahrami and Andrew, the 2-year OS rate of FS patients was <70%, while that for 5-year was <55% [14].

Since FS is a malignant tumor, does a giant tumor predict a poor prognosis? According to the researches of Zhang et al. and Sulkowski et al., tumor size had no differential influence on the survival prognosis of FS patients with ovarian and infantile, respectively [18, 19]. However, Ma et al. found that a tumor size more than 5 cm and a Ki-67 index over 30% were associated with poor OS in patients with primary intracranial FS [20]. Furthermore, according to a study by Xiang et al., tumor size was the most important factor that determines CSS in FS patients [15]. The possible explanation is that there are certain differences in the conclusions drawn for a specific population, the location of the disease, and the number of enrolled patients, but they can also assist in disease management. Our research also concluded that tumor size was associated with the CSS of POFS patients based on a larger number of patients, so it is meant to include tumor size in the survival prediction of POFS patients. In addition, tumor grade is closely associated with the prognosis of soft tissue sarcoma. According to reports, the 10-year mortality rate for low-grade tumors is 40%, while that for high-grade tumors is as high as 70% [2]. We have also reached a similar conclusion that patients with high-grade tumors have a worse prognosis than patients with low-grade tumors. At the same time, the SEER stage is also closely associated with the prognosis of soft tissue sarcoma. The prognosis of patients with distant metastasis is significantly worse than that of local or regional metastasis, illustrating the importance of early diagnosis and surgical intervention for FS patients.

Adjuvant radiotherapy and chemotherapy for soft tissue sarcoma are controversial and are not standard treatments for these tumors [21]. Andrew L. Folpe found that almost 80% of adult FSs were high-grade (FNCLCC grade 2–3) malignant tumors, with one out of every four low-grade lesions progressing to high-grade sarcoma in local recurrence [14]. At the same time, 9–63% of adult FS patients who had hematological spread and metastasis, including patients whose tumors were fully resected, would experience recurrence, resulting in a poor prognosis [4, 9]. Although radiotherapy and chemotherapy have a low response rate for FS, they are still utilized as neoadjuvant/adjuvant tumor therapy, and patients with high-grade FS who are at risk of metastasis are the most likely to benefit from adjuvant therapy [13, 22]. Muehlhofer et al. published a retrospective single-center investigation spanning more than 15 years, which showed that soft tissue sarcoma patients had a 5-year OS rate of 68.9%. Radiation therapy type (adjuvant or neoadjuvant radiation therapy) had no influence on survival [23]. However, data from 6,960 soft tissue sarcoma patients in the SEER database suggested that radiotherapy was related to the improved survival rate of high-grade tumors patients [24]. Augsburger et al. believed that for FS with deep location, high-grade, and tumor size beyond 5 cm, performing radiotherapy after R0 resection was strongly recommended. However, the necessity of adjuvant radiotherapy has not yet been determined for other types of tumor classification, size, and location [6, 25]. As for chemotherapy, it targets and kills rapidly dividing and proliferating cells, such as malignant tumor cells. Doxorubicin and other chemotherapy drugs are the primary drugs used for those patients. Ma et al. found that giving Apatinib to recurrent FS patients with high VEGF-2 expression could provide short-term benefits and reduce the incidence of adverse reactions. However, the number of FS patients who do not respond well to chemotherapy was large, limiting the therapeutic effectiveness of chemotherapy drugs [26]. Interestingly, in our study, chemotherapy and radiotherapy were not independent prognostic indicators of CSS in POFS patients.

The SEER database has sufficient sample data of cancer patients to ensure the reliability of the research conclusions. No nomogram, however, can precisely quantify the impact of predictors on prognosis 100% of the time. There are some drawbacks in our nomogram: (i) selection bias is unavoidable in a clinical retrospective study; (ii) the tumor size is a crucial variable included in our study, but the tumor size has only been recorded in the SEER database since 2004, shortening the data period and the number of patients. Therefore, a longer time frame and larger population may help further to improve the credibility and persuasiveness of model predictions. At the same time, the lack of some essential variables in the SEER database also limits the use of the model, such as surgical margin status; (iii) more data from other research centers for external verification will increase the applicability and accuracy of the new nomogram.

## 5. Conclusion

In conclusion, soft tissue sarcomas involving FS are a rare and aggressive variety. In the management of FS patients, surgical resection is crucial. By analyzing 346 POFS patients with complete data in the SEER database, it is concluded that tumor size, SEER stage, and tumor stage are independent prognostic predictors for CSS in POFS patients. Based on the three variables, a nomogram and a risk classification system were built to predict the 1-, 3-, and 5-year CSS of POFS patients. They were helpful for the treatment decision-making, surveillance, and counseling, thus maximizing the benefit of POFS patients.

## Figures and Tables

**Table 1:** The baseline characteristics of the CSS-related variables of postoperative fibrosarcoma patients.

Variables	Training set		Validation set		Total	
	244	70.00 (%)	102	30.00 (%)	346	100.00 (%)
Age (years old)						
<43	98	40.16	39	38.23	137	39.60
43–71	123	50.41	49	48.04	172	49.71
>71	23	9.43	14	13.73	37	10.69

Sex						
Male	126	51.64	60	58.82	186	53.76
Female	118	48.36	42	41.18	160	46.24

Marital status						
Unmarried	127	52.05	47	46.08	174	50.29
Married	117	47.95	55	53.92	172	49.71

Race						
Black	49	20.08	26	25.49	75	21.67
White	176	72.13	61	59.80	237	68.50
Other	19	7.79	15	14.71	34	9.83

Tumor size (mm)						
<64	150	61.48	66	64.71	216	62.42
64–110	52	21.31	24	23.53	76	21.97
>110	42	17.21	12	11.76	54	15.61

SEER stage						
Localized	185	75.82	84	82.35	269	77.74
Regional	49	20.08	15	14.71	64	18.50
Distant	10	4.10	3	2.94	13	3.76

Tumor grade						
Grade I	100	40.98	30	29.41	130	37.57
Grade II	71	29.10	33	32.35	104	30.06
Grade III	40	16.39	21	20.59	61	17.63
Grade IV	33	13.53	18	17.65	51	14.74

Radiotherapy						
No	162	66.40	69	67.65	231	66.76
Yes	82	33.60	33	32.35	115	33.24

Chemotherapy						
No	212	86.90	94	92.16	306	88.44
Yes	32	13.10	8	7.84	40	11.56

**Table 2:** The univariate and multivariate Cox regression analyses of the CSS-related variables of postoperative fibrosarcoma patients.

Variables	Univariate analysis		Multivariate analysis	
	Hr (95% CI)	*p* value	Hr (95% CI)	*p* value
Age (years)				
<43	References			
43–71	2.873 (1.294–6.379)	0.01		
>71	6.866 (2.755–17.113)	≤0.001		

Sex				
Male	References			
Female	0.667 (0.367–1.214)	0.185		

Marital status				
Unmarried	References			
Married	1.376 (0.758–2.499)	0.295		

Race				
Black	References			
White	3.341 (1.032–10.814)	0.044		
Other	1.597 (0.267–9.563)	0.608		

Tumor size (mm)				
<64	References		References	
64–110	4.016 (1.692–9.533)	0.002	3.997 (1.640–9.745)	0.002
>110	11.567 (5.347–25.025)	≤0.001	8.140 (3.624–18.279)	≤0.001

SEER stage				
Localized	References		References	
Regional	2.544 (1.130–4.939)	0.006	1.549 (0.751–3.195)	0.236
Distant	10.234 (4.528–23.133)	≤0.001	4.598 (1.929–10.962)	≤0.001

Tumor grade				
Grade I	References		References	
Grade II	9.815 (2.230–43.193)	0.003	9.844 (2.229–43.480)	0.003
Grade III	17.895 (4.066–78.755)	≤0.001	11.704 (2.631–52.056)	≤0.001
Grade IV	24.892 (5.655–109.574)	≤0.001	16.751 (3.731–75.206)	≤0.001

Radiotherapy				
No	References			
Yes	2.811 (1.532–5.159)	≤0.001		

Chemotherapy				
No	References			
Yes	4.505 (2.453–8.274)	≤0.001		

## Data Availability

Publicly available datasets were analyzed in this study. These data can be found in the SEER dataset repository (https://seer.cancer.gov/).

## Abbreviations

SEER: Surveillance, Epidemiology, and end results
POFS: Postoperative fibrosarcoma
WHO: World Health Organization
DFSP: Dermatofibrosarcoma protuberans
OS: Overall survival
CSS: Cancer-specific survival
ROC: Receiver operating characteristic
DCA: Decision curve analysis
AUCs: Area under the curves
TNM: Tumor-node-metastasis.

## Supplementary Materials

Supplementary Materials Supplementary Figure 1: according to the X-tile software, the best cut-off values for the age were determined to be 43 and 71 (years). Supplementary Figure 2: according to the X-tile software, the best cut-off values for the tumor size were determined to be 64 and 110 (mm). Supplementary Figure 3: according to the X-tile software, the best cut-off values for the overall survival score were determined to be 87 and 156. Table S1: the values assigned to CSS-related variables in our study. Table S2: the detailed scores of independent prognostic factors in the CSS nomogram.
