# Supplementary material for: A Novel Prognostic Nomogram and Risk Classification System for Predicting Cancer-Specific Survival of Postoperative Fibrosarcoma Patients: A Large Cohort Retrospective Study
Source: J Oncol. 2022 Aug 27;2022:7831001. doi: 10.1155/2022/7831001 (PMC9440790; doi:10.1155/2022/7831001)
Supplement: Supplementary Materials — Supplementary Figure 1: according to the X-tile software, the best cut-off values for the age were determined to be 43 and 71 (years). Supplementary Figure 2: according to the X-tile software, the best cut-off values for the tumor size were determined to be 64 and 110 (mm). Supplementary Figure 3: according to the X-tile software, the best cut-off values for the overall survival score were determined to be 87 and 156. Table S1: the values assigned to CSS-related variables in our study. Table S2: the detailed scores of independent prognostic factors in the CSS nomogram. [file 7831001.f1.zip › Supplementary file-1-R1.docx]

**Supplementary figures**


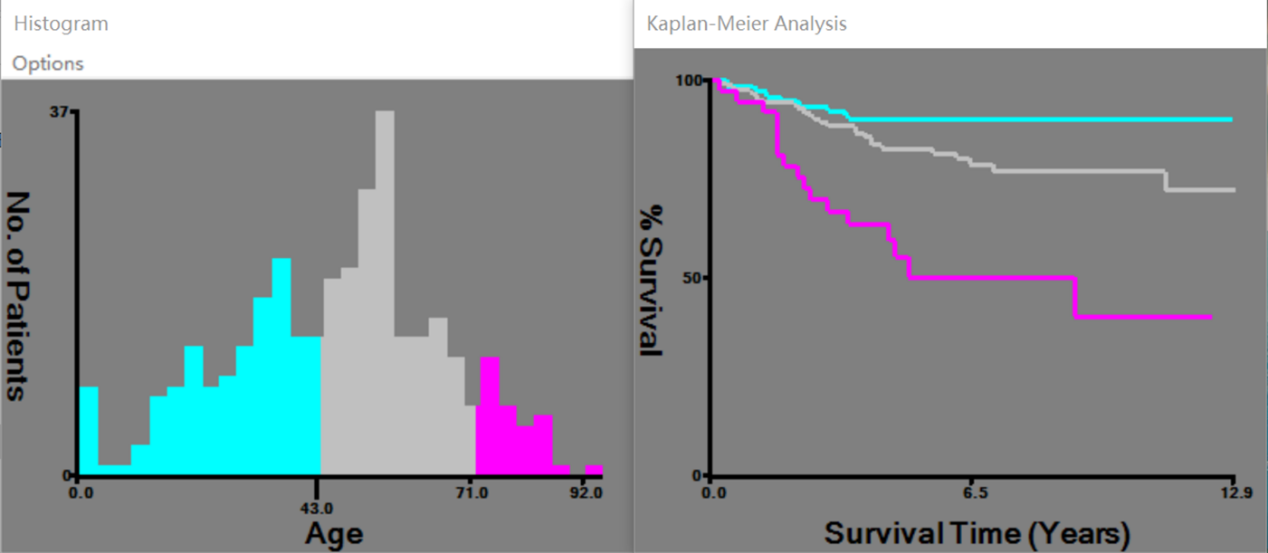


**Supplementary figure 1:** According to the X-tile software, the best cut-off values for the age were determined to be 43 and 71 (years).


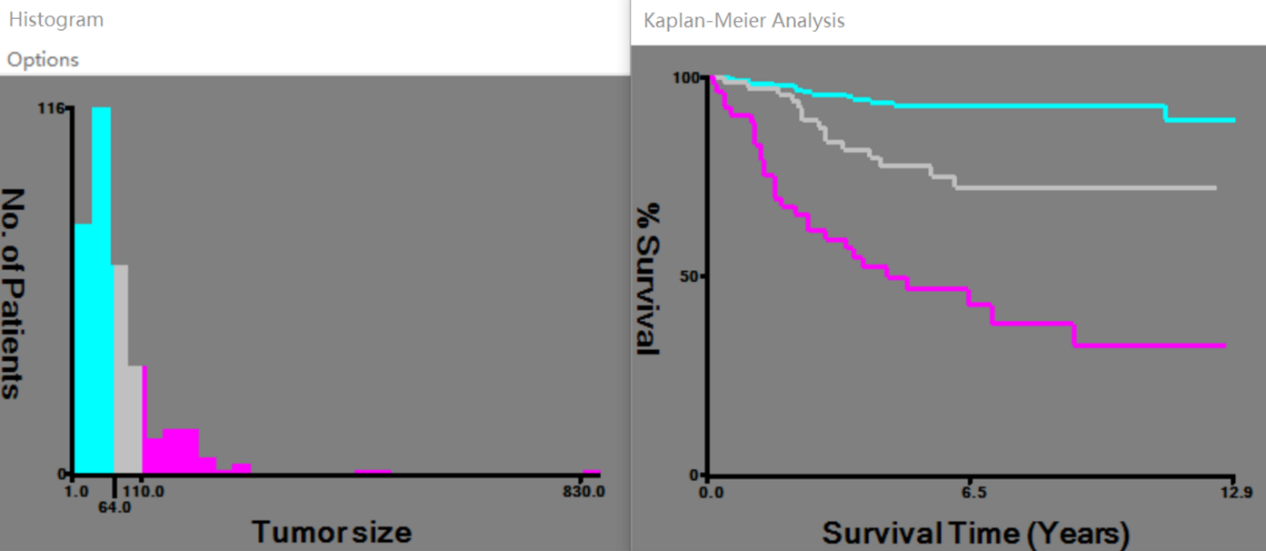


**Supplementary figure 2:** According to the X-tile software, the best cut-off values for the tumor size were determined to be 64 and 110 (mm).


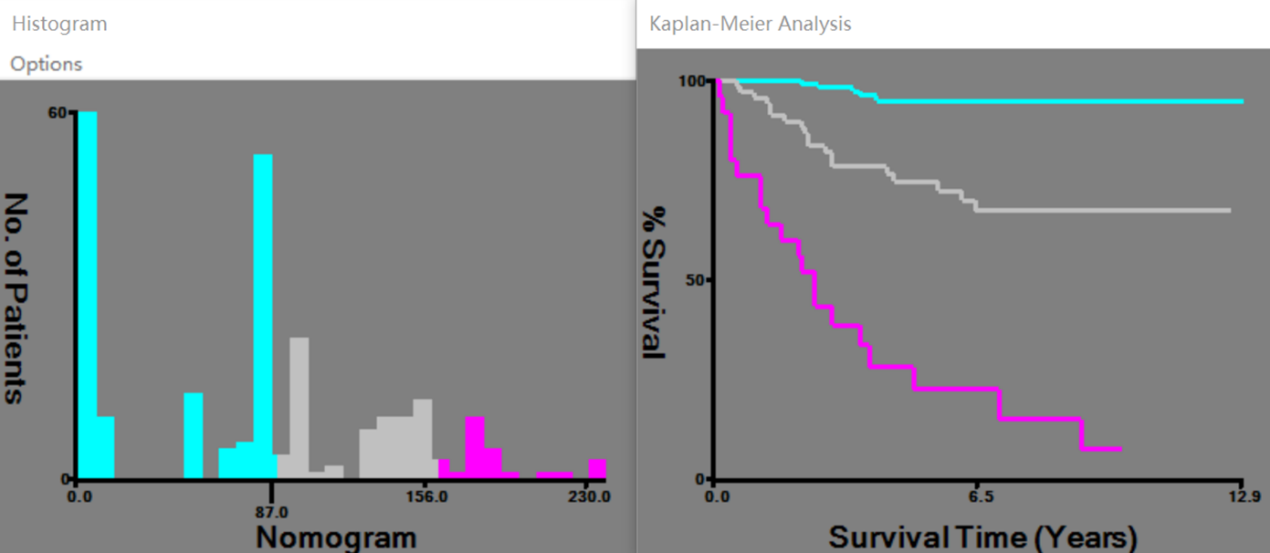


**Supplementary figure 3:** According to the X-tile software, the best cut-off values for the overall survival point were determined to be 87 and 156.
