# Supplementary material for: A Novel Prognostic Nomogram and Risk Classification System for Predicting Cancer-Specific Survival of Postoperative Fibrosarcoma Patients: A Large Cohort Retrospective Study
Source: J Oncol. 2022 Aug 27;2022:7831001. doi: 10.1155/2022/7831001 (PMC9440790; doi:10.1155/2022/7831001)
Supplement: Supplementary Materials — Supplementary Figure 1: according to the X-tile software, the best cut-off values for the age were determined to be 43 and 71 (years). Supplementary Figure 2: according to the X-tile software, the best cut-off values for the tumor size were determined to be 64 and 110 (mm). Supplementary Figure 3: according to the X-tile software, the best cut-off values for the overall survival score were determined to be 87 and 156. Table S1: the values assigned to CSS-related variables in our study. Table S2: the detailed scores of independent prognostic factors in the CSS nomogram. [file 7831001.f1.zip › Supplementary file-2 (1).docx]

**Table S1.** The values assigned ​​to CSS-related variables in our study.

| **CSS-related variables** | **Assigned values** |
| --- | --- |
| **Age (years)** | |
| ＜43 | 0 |
| 43-71 | 1 |
| ＞71 | 2 |
| **Sex** | |
| Male | 0 |
| Female | 1 |
| **Marital status** | |
| Unmarried | 0 |
| Married | 1 |
| **Race** | |
| Black | 1 |
| White | 2 |
| Other | 3 |
| **Tumor size (mm)** | |
| ＜64 | 1 |
| 64-110 | 2 |
| ＞110 | 3 |
| **SEER stage** | |
| Localized | 1 |
| Regional | 2 |
| Distant | 3 |
| **Tumor** **grade** | |
| Grade I | 0 |
| Grade II | 1 |
| Grade III | 2 |
| Grade IV | 3 |
| **Radiotherapy** | |
| No | 0 |
| Yes | 1 |
| **Chemotherapy** | |
| No | 0 |
| Yes | 1 |
